# Social relationship satisfaction and PTSD: which is the chicken and which is the egg?

**DOI:** 10.3402/ejpt.v6.28864

**Published:** 2015-12-16

**Authors:** Sara A. Freedman, Moran Gilad, Yael Ankri, Ilan Roziner, Arieh Y. Shalev

**Affiliations:** Center for Traumatic Stress Studies, Hadassah University Hospital, Jerusalem, Israel

**Keywords:** Social relationship satisfaction, PTSD, natural recovery

## Abstract

**Background:**

Impaired social relationships are linked with higher levels of posttraumatic stress disorder (PTSD), but the association's underlying dynamics are unknown. PTSD may impair social relationships, and, vice versa, poorer relationship quality may interfere with the recovery from PTSD.

**Objective:**

This work longitudinally evaluates the simultaneous progression of PTSD symptoms and social relationship satisfaction (SRS) in a large cohort of recent trauma survivors. It also explores the effect of cognitive behavior therapy (CBT) on the association between the two.

**Method:**

Consecutive emergency department trauma admissions with qualifying PTSD symptoms (*n=*501) were assessed 3 weeks and 5 months after trauma admission. The World Health Organization Quality of Life evaluated SRS and the Clinician Administered PTSD Scale evaluated PTSD symptom severity. Ninety-eight survivors received CBT between measurement sessions. We used Structural Equation Modeling to evaluate cross-lagged effects between the SRS and PTSD symptoms.

**Results:**

The cross-lagged effect of SRS on PTSD was statistically significant (β=−0.12, *p*=0.01) among survivors who did not receive treatment whilst the effect of PTDS on SRS was nil (β=−0.02, *p*=0.67). Both relationships were non-significant among survivors who received CBT.

**Discussion:**

SRS and PTSD are highly associated, and this study shows that changes in SRS in the early aftermath of traumatic events contribute to changes in PTSD, rather than vice versa. SRS impacts natural recovery, but not effective treatment. This study suggests that being satisfied with one's relationships might be considered as an important factor in natural recovery from trauma, as well as in intervention.

Difficulties in social relationships, including relationship quality, satisfaction, intimacy, cohesion, and sexual satisfaction, have all been associated with the presence of posttraumatic stress disorder (PTSD) in one or both partners (Dekel, Enoch, & Solomon, 2008; Koenen, Stellman, Sommer, & Stellman, 2008; Lunney & Schnurr, 2007). This association, although well established, is not well understood.

Social relationships comprise many different factors and can be assessed from different standpoints. One aspect is satisfaction with intimate social relationships, usually marital partners. Another refers to satisfaction with social roles, such as parenting. A further factor is related to behavior patterns, such as within a marriage. Social support is a particular facet of social relationships, examining perceived readiness of others to provide help in times of need. Clearly, these different factors overlap, such that higher perceived social support from a spouse is likely related to reported satisfaction in that relationship, as well as being related to nurturing behaviors within that relationship (Cundiff, Smith, Butner, Critchfield, & Nealey-Moore, 2015). However, most studies have examined these factors separately.

Two conflicting hypotheses exist regarding the role of social relationships in PTSD. On the one hand, PTSD may lead to poorer social relationships. There is some support for this notion. First, studies have shown that its particularly numbing symptoms have been shown to be negatively related to relationship satisfaction (Campbell & Renshaw, 2013). Second, two prospective studies with military populations have examined the association of relationships and PTSD, one showing that PTSD results in poorer relationships (Campbell & Renshaw, 2013), and the other that increases in PTSD results in a detrimental effect on marital relationships and parenting (Gewirtz, Polusny, DeGarmo, Khaylis, & Erbes, 2010).

Third, studies examining PTSD and social support have shown that initial PTSD levels predict later social support in military populations (e.g., King, Taft, King, Hammond, & Stone, 2006; Solomon & Mikulincer, 1990).

These studies seem to indicate therefore that PTSD has detrimental effects on social relationships. The opposing hypothesis that poorer social relationships, which increase the likelihood of developing PTSD, also has some support in the literature, because the social support in general has been shown to be a consistent predictor of PTSD (Andrews, Brewin, & Rose, 2003). Studies that have examined PTSD and relationship and role satisfaction, and behavior, have not, to our knowledge, examined the possibility that poorer relationships may lead to PTSD. Indeed, the studies described above that examined relationship satisfaction rather than social support, assessed relationship satisfaction only at the second time point, and therefore the hypothesis that PTSD might be subsequent to poorer relationships could not be tested.

One longitudinal study has shown that both these opposing hypotheses may be correct, such that perceived social support amongst family members is a predictive factor of PTSD soon after a traumatic event, but over time, this relationship is reversed, and PTSD levels then predict social support (Kaniasty & Norris, 1993).

A related area of research is the effect that social relationships have on recovery from PTSD. A small number of studies have shown that greater social support (Thrasher, Power, Morant, Marks, & Dalgleish, 2010) and lower expressed emotion (Tarrier, Sommerfield, & Pilgrim, 1999) are related to recovery from chronic PTSD. However, the impact of social relationships has not been considered as a potential factor in early recovery. The first months after trauma exposure are critically important for the development of persistent PTSD. Indeed, most survivors who meet initial PTSD diagnostic criteria recover from PTSD within 1 year (Freedman, Brandes, Peri, & Shalev, 1999; Kessler, 2000). It is plausible that qualities of interpersonal relationships may critically affect the likelihood of recovery early after a traumatic event, either spontaneously or with treatment. To our knowledge, no study has previously examined this relationship.

To address this gap, we here present a novel analysis of data from a randomized control study, in which patients were evaluated at multiple time points during the months that followed trauma exposure (Shalev et al., 2011, 2012). In addition to symptom levels at all time points, subjects’ perceptions of their social relationships were also assessed. This measure assesses three of the social relationship factors described above: perceived social support, perceived satisfaction with social relationships in general, and perceived satisfaction with intimate relationships. Specifically, we use these data to address two alternative hypotheses: decreased social relationship satisfaction (SRS) would be associated with subsequent higher levels of PTSD, and that increased PTSD would be associated with subsequent poorer SRS, in both cases controlling for treatment type.

## Method

### Participants

Participants were 501 individuals who had attended the emergency room following a civilian trauma meeting Criterion A of DSM (American Psychiatric Association, 2000). Women were 49.7% of the sample, men were 50.3%. The mean participants’ age was 36.22 (*SD=*11.84) and it ranged from 20 to 69. SES was measured on a 5-point scale, ranging from very low (1) to very high (5); the mean SES was 2.4 (*SD*=1.1).

### Measures

#### Clinical interviews

*Clinician Administered PTSD Scale (CAPS; Blake et al., 1995*): PTSD was assessed at both time points using the CAPS. The CAPS gives a score for both frequency and intensity of the 17 PTSD symptoms (DSM IV), and a continuous score is calculated by summing all these. In this study, Cronbach's α was 0.93 at Time I and 0.96 at Time II.

*SCID Structured Clinical Interview for DSM-IV, (First, Spitzer, Gibbon, & Williams, 1997*): This was used to screen for all current and past Axis I disorders. In this data set, *past depression* was measured using the SCID at Clinical Interview I. It was dummy-coded with 1=yes.

*SRS was measured using the* World Health Organization Quality of Life-*BREF (Group, 1998*): This is the 26-item version of the original questionnaire and measures subjective quality of life, in four dimensions: physical health, psychological health, social relationships, and environment. Responses are measured on a 5-point scale, with a higher number indicating a better perceived quality of life. In this study, the three questions from the social relationships section were included: How satisfied are you with your personal relationships?; How satisfied are you with your sex life?; and How satisfied are you with the support you get from your friends? In this study, Cronbach's α was 0.75 at Clinical Interview I and 0.81 at Clinical Interview II.

*Negative life events* (NLE) were measured using the Stressful Life Events Screening Questionnaire—SLESQ (Goodman, Corcoran, Turner, Yuan, & Green, 1998); this is the Hebrew version of the SLESQ, used in several previous studies. A total number of endorsed events were used.

*Sociodemographic variables* were (1) sex (dummy-coded, with male=1); (2) age in years; (3) schooling years; (4) marital status (dummy-coded, with married=1 and otherwise=0); (5) number of children (0–9); (6) household density (defined as the number of household members divided by the number of rooms in the household); and (7) self-reported income (based on participants’ responses to a 5-point scale ranging from 1=way below average to 5=way above average).

*Trauma type*, with two types (MVA and Terror) as the dummy-coded variables, and other types serving as the base.

*Randomized control trial* (RCT) condition was expressed as two dummy-codes of WL and PE/CT, with non-RCT serving as the base.

### Procedure

This study examined data collected as part of the Jerusalem Trauma Outreach and Prevention Study (J-TOPS; ClinicaltrialsGov Identifier: NCT0014690); the study included systematic outreach and follow-up of recent trauma survivors, as well as an embedded randomized controlled study examining the effects of early interventions for preventing chronic PTSD. The study's procedures have been fully described previously (Shalev et al., 2011) and will be briefly described here.

Subjects were adults aged 18–70 who were consecutive trauma survivors attending the emergency room of a level I trauma center. Traumatic events were mixed civilian events, with the majority (80%) being motor vehicle accidents. Eligible participants were recruited via a telephone interview 10 days following the event (*N=*4,743) and gave verbal informed consent. During this initial telephone interview, participants who had experienced a traumatic event according to criteria A1 and A2, and met other study criteria (*N=*1,996) were assessed for initial symptoms of acute stress disorder. Participants with acute PTSD symptoms (*N=*1,502) were invited to attend a clinical interview 3 weeks post trauma, of which 756 attended. At this clinical interview (Clinical Interview I), subjects signed written informed consent and were assessed for current and lifetime psychiatric disorders using the SCID, and for the presence of PTSD symptoms. Three hundred ninety-seven subjects showed sufficient symptoms (acute PTSD minus the time criterion) and were invited to participate in the randomized controlled trial. Two hundred ninety-six subjects agreed to enter the trial and were randomized using equipoise stratified randomization to one of five treatment arms: prolonged exposure (PE, *N=*63), cognitive therapy (CT, *N=*40), SSRI (*N*=23), placebo (*N=*23), and waiting list control (*N=*93). Results indicated that PE and CT showed similar effectiveness, and for the purposes of these analyses are considered one group, namely PE/CT. All patients received 12 weeks of treatment.

As described above, the RCT was embedded in a follow-up study of all subjects, and as such participants (*N=*756) who attended the first clinical interview were reassessed 5 months after the trauma (*N=*604, Clinical Interview II), regardless of participation in the RCT. Assessors were blind to participation in the RCT. Hadassah University Hospital's Institutional Review Board approved and monitored the study.

The data used in this analysis concentrate on 501 individuals who have available data at the pre- and post-early treatment clinical assessments; that is, at 3 weeks and 5 months post trauma, Clinical Interviews I and II. Three groups were included: those who received either PE or CT (PE/CT, *N=*98); those who were in the waiting list control group (WL, *N=*90); and those who did not enter the RCT and therefore did not receive any treatment (non-RCT, *N=*313). It is important to include this non-treatment group, because it represents an understudied population, and it is rare to have data that allow for comparison of subjects who did and did not enter an RCT. The SSRI/Placebo group was small and had insufficient numbers at follow-up to be included in the present analyses. SRS and PTSD symptom levels were assessed at both time points.

### Data analytic plan

The main analysis approach was Structural Equation Modeling, done with the Mplus 7.11 program (Muthén & Muthén, 2012). The minimal covariance coverage in the variance–covariance matrix used in the analyses was 0.83. To take advantage of all the available data, models were fit using full-information maximum likelihood estimation with robust standard errors (Little & Rubin, 2003). Following recommendations of Hu and Bentler (1999), we report fit indexes of two types: the Tucker–Lewis index (TLI) and the Comparative Fit Index (CFI), and two indexes of misfit: root mean-square error of approximation (RMSEA) and standardized root mean-square residual (SRMR). NNFI and CFI close to or above 0.95, combined with RMSEA below 0.06 and SRMR below 0.08, are considered indicative of acceptable fit. SRS was specified as a latent variable, indicated by its three items. PTSD was also specified as a latent variable, measured with three indicators, each created as a random third of the scale items using the accepted approach of parceling (Bandalos, 2002; Stacy, Bentler, & Flay, 1994). To test for reciprocal causal associations between SRS and PTSD, we fit, within the SEM framework, a cross-lagged panel model (Finkel, 1995). Such models allow for testing the causal associations of two variables while considering for their stabilities and controlling for measuring issues. For this, we allowed for measurement errors of the same indicators to correlate over time in autoregressive paths and fixed factor loadings to equality over time.

## Results

Descriptive statistics of observed research variables and their correlations with relationship satisfaction and PTSD measures are provided in Table 1.

**Table 1:** Means and standard deviations for the research variables and their correlations with SRS and PTSD

Variable	Mean	*SD*	Social relationship satisfaction T1	Social relationship satisfaction T2	PTSD T1	PTSD T2
RCT group: waiting list^a^	0.18	0.38	−0.09	−0.18***	0.22***	0.29***
RCT group: PE/CT^a^	0.20	0.40	−0.16***	−0.02	0.24***	−0.01
Sex: male^a^	0.50	0.50	0.02	0.06	0.02	−0.05
Age (in years)	36.22	11.84	−0.06	−0.16***	−0.04	0.11*
Education (in years)	13.19	2.67	0.00	0.00	0.00	0.04
Marital status: married^a^	0.51	0.50	0.00	−0.05	0.02	0.06
Number of children	1.60	1.98	−0.04	−0.08	0.06	0.11*
Household density	1.05	0.59	−0.02	−0.06	−0.07	−0.06
Income	2.40	1.10	0.09*	0.08	−0.13**	−0.01
Negative life events	1.80	1.77	−0.13**	−0.17***	0.05	0.07
Past depression^a^	0.27	0.44	−0.10*	−0.16***	0.13**	0.05
Trauma type: terror^a^	0.13	0.33	−0.01	0.02	0.09*	0.05
Trauma type: MVA^a^	0.83	0.37	−0.01	−0.02	−0.06	−0.02
Social relations T1	3.29	0.96	—			
Social relations T2	3.50	0.97	0.58***	—		
PTSD T1	1.58	0.73	−0.40***	−0.29***	—	
PTSD T2	0.81	0.74	−0.32***	−0.52***	0.57***	—

MVA, motor vehicle accident; Terror, terrorist attack.

* *p<*0.05;

** *p<*0.01;

*** *p<*0.001.

a Dummy-coded variable, 1=the value specified in the variable name, 0=other.

As can be seen in Table 1, PTSD symptoms are negatively correlated with SRS, at both time points. Higher frequency of NLE and higher levels of past depression are associated with lower SRS at both time points. In addition, age is negatively correlated with SRS and positively correlated with PTSD, both at T2. Participants who reported higher income also reported better RS and lower PTSD at T1, but not at T2.

**Fig. 1:**
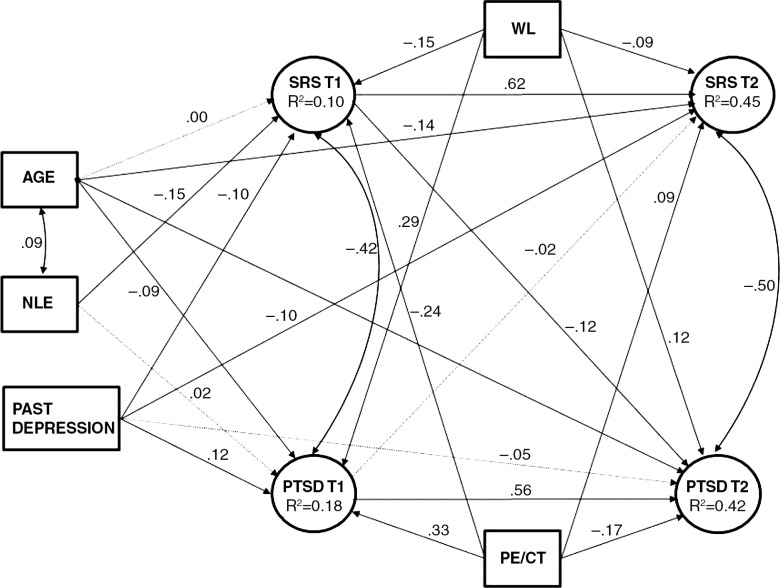
Structural equation model of cross-lagged relationship satisfaction and PTSD effects with standardized parameters. *χ* ^2^ (92, *N*=501)=158.92, *p<*0.0001, TLI=0.984, CFI=0.988, SRMR=0.037, and RMSEA=0.037. The solid lines indicate paths statistically significant at *p<*0.05. The dotted lines indicate non-significant paths. NLE, negative life effects; SRS, social relationship satisfaction; WL, waiting list; PE/CT, prolonged exposure/cognitive therapy.

As the first stage of the main analyses, we tested the measurement model. It yielded acceptable results: *χ* ^2^ (46, *N*=501)=66.26, *p*=0.03, TLI=0.995, CFI=0.996, SRMR=0.030, RMSEA=0.030 (90% CI=0.010; 0.045). We proceeded then to test the cross-lagged panel model. To this model, we added the two dummy variables expressing membership in the two RCT experimental groups as predictors of both T1 and T2 relationship satisfaction and PTSD. Parceling out the group membership from T1 measures allowed us to account for the initial imbalance in the groups’ composition. The group membership effects upon the T2 measures controlled for T1 measures allow us to estimate the RCT impact upon the change over time in these measures. We also added to the model, as predictors of each of the four content variables, those sociodemographic and background variables that were correlated with any of the content research variables (Table 1). This structural model fit the data well, with *χ* ^2^ (114, *N*=501)=208.80, *p<*0.0001, TLI=0.978, CFI=0.983, SRMR=0.039, and RMSEA=0.041 (90% CI=0.032; 0.049). In this model, all the paths emitted from two control variables, number of children and self-reported income, were not statistically significant. These variables were deleted from the model. The paths from NLE to relationship satisfaction and PTSD at T2 were also non-significant and were therefore fixed to zero. The resulting model (Fig. 1) showed good fit to the data, with *χ* ^2^ (92, *N*=501)=158.92, *p<*0.0001, TLI=0.984, CFI=0.988, SRMR=0.037, and RMSEA=0.037 (90% CI=0.027; 0.047).

As seen in Fig. 1, both SRS and PTSD exhibited some stability across time (with stability coefficients of 0.62 and 0.56, accordingly). The correlations between them were rather high, both at T1 (*r*=−0.42) and T2 (*r*=−0.50). Importantly, the cross-lagged effect of PTSD upon SRS was close to zero (β=−0.02, *p*=0.67), whereas the parallel effect of SRS upon PTSD was small in magnitude, but statistically significant (β=−0.12, *p*=0.01). This pattern of results is consistent with the hypothesis that initial levels of SRS are the driving force in the development of PTSD.

At the next step, we added to the model the interactive effects of RCT group membership, WL, or PE/CT, and the initial level of each of the focal research variables, SRS or PTSD, upon the other variable at T2. To obtain a clear picture, we tested each of these effects within separate models. Three out of four interactive effects failed to reach statistical significance (for WL×PTSD, *p*=0.66; for PE/CT×PTSD, *p*=0.63; and for WL×SRS, *p*=0.11). The interaction of WL×SRS was significant, β=0.19*, p*=0.02. To illustrate the form of this interaction, the cross-lagged model was fit separately for the three experimental groups. The path from T1 relationship satisfaction to T2 PTSD was negative and significant in two groups, non-RCT (β=−0.18*, p<*0.05) and WL (β=−0.26*, p<*0.05), but was not significant in the PE/CT group (β=0.12*, p>*0.30).

## Discussion

To the best of our knowledge, this is the first prospective analysis examining SRS, PTSD, and treatment response in a civilian population. The results indicate that increased PTSD symptoms are correlated with decreased SRS, and this corroborates previous studies (Campbell & Renshaw, 2013; Gewirtz et al., 2010).

However, the results of the path analysis indicate that poorer SRS may drive PTSD symptoms, rather than the reverse. Previous studies (Campbell & Renshaw, 2013; Gewirtz et al., 2010) have shown that PTSD results in decreased satisfaction, but as noted above, these studies did not examine initial relationship satisfaction. These results imply that patients who present with trauma exposure and relationship difficulties may be particularly vulnerable to PTSD development. It is possible that the discrepancy with previous studies arises from the different populations studied—civilian as opposed to military. As has been noted (De Burgh, White, Fear, & Iversen, 2011), the marital relationship is placed under particular strain in military families, due in part to long absences and frequent moving, and therefore it may not be relevant to compare these populations.

In survivors who received treatment, the significant relationship between decreased satisfaction with relationships and elevated PTSD is not apparent, whereas it remains significant in those groups who did not receive treatment, either within the RCT (waiting list) or those who did not enter the treatment trial. These results do not support previous studies showing that greater social support is related to better treatment response (Thrasher et al., 2010). This may be explained by the timing of assessments in the current study. Previous studies have all examined the treatment or effects of chronic PTSD, rather than preventative treatment early after a traumatic event. Patients in the current study had suffered from symptoms for a relatively short period of time (5 weeks) before beginning treatment. This may not be long enough for the adverse effects of PTSD on previously satisfying relationships to be felt, and therefore effects on treatment response may not be seen.

The current results may indicate that the process of natural recovery is enhanced when the individual reports satisfaction with relationships, but impaired with poorer satisfaction. Treatment appears to amend this relationship, perhaps showing that when an intervention is successful at impacting PTSD levels, the levels of relationship satisfaction is immaterial to outcome. This may also be attributed to a therapeutic relationship that compensates for the lack of satisfying social relationships (Beutler, Forrester, Holt, & Stein, 2013).

Taken together, these results indicate that relationship satisfaction may play a part in the development of PTSD, and natural recovery from it. Chronic PTSD is associated with marital difficulties and parenting problems (Dekel & Monson, 2010) which in themselves can lead to traumatization of other family members (Berz, Taft, & Watkins, 2008). The results presented here may show that even though early treatment has little long-term benefit over later treatment (Shalev et al., 2012) it may positively impact family relationships and this has beneficial effect beyond PTSD symptoms.

This study is limited first by the measurement of relationship satisfaction. Recent studies examining family interactions have preferred to use multiple assessments, thus gaining input, for example, from both partners in a relationship (Holmbeck, Li, Schurman, Friedman, & Coakley, 2002) as opposed to self-report measures of one partner. The second limitation regards the sample, consisting of one-time traumatic events in a civilian population. This analysis should be replicated in other trauma populations.

## Conclusions

These novel results indicate that the impact of PTSD and social relationships on each other needs to be more fully explored. Recent clinical trials have shown the importance of including significant others in PTSD treatment (Monson et al., 2012), and these results support this approach. Future studies might systematically assess social relationships, their impact on treatment, and the impact that treatment (or lack of it) has on them.

## Supplementary Material

Social relationship satisfaction and PTSD: which is the chicken and which is the egg?
