# Supplementary material for: Social relationship satisfaction and PTSD: which is the chicken and which is the egg?
Source: Eur J Psychotraumatol. 2015 Dec 16;6:10.3402/ejpt.v6.28864. doi: 10.3402/ejpt.v6.28864 (PMC4696463; doi:10.3402/ejpt.v6.28864)
Supplement: Social relationship satisfaction and PTSD: which is the chicken and which is the egg? [file EJPT-6-28864-s001.pdf]

Związek pomiedzy poczyciem satysfakcji i PTSD: co było pierwsze, jajko czy kura?

Sara A Freedman, Moran Gilad, Yael Ankri, Ilan Roziner, Arieh Shalev

Wprowadzenie: Istnieje wiele badań wskaujących na dodatki związek pomiedzy nasileniem objawów PTSD, a zubożeniem relacji społecznych. Nie jest jasny natomiast kierunek tego związku, tj. nie do końca wiadomo, czy objawy PTSD zubożają relacje społeczne, czy też jest odwrotnie, tj. brak satysfakcji z relacji społecznych nasila objawy PTSD.
Cel: Celem niniejszej pracy była analiza związku pomiędzy nasileniem objawó PTSD i satysfkacją z relacji społecznych na dużej próbie osób po traumie w schemacie badan podłużnych.
Metoda: Zbadano 501 osób po doswiadczeniu różnego rodzaju doswiadczeń traumatycznych, przy uzyciu skali CAPS do pomiaru objawów PTSD oraz kwestioanriusza WHQ-QOL do analizy satysfakcji z relacji społecznych. Cześć badanych otrzymywała wspracie w postaci psychoterapii.
Wyniki i dyskusja : Wyniki niniejszych badań wykazały, że satysfakcja z relacji społecznych i PTSD są silnie ze soba związane oraz że zmiany w poziomie satysfakcji z relacji społecznych mogą przyczynić się do zmian w nasileniu PTSD, ale nie odwrotnie. Badanie to pokazao zatem, ze satysfkacja z relacji społecznych może być ważnym czynnikiem chroniacym przed objwami PTSD osoby po traumie.

Keywords:

Name of translator: Marcin Rzeszutek, University of Finance and Management in Warsaw, Poland

Citation: European Journal of Psychotraumatology 2015, **6**: 28864 - http://dx.doi.org/10.3402/ejpt.v6.28864
